# Comparison of Metformin and Repaglinide Monotherapy in the Treatment of New Onset Type 2 Diabetes Mellitus in China

**DOI:** 10.1155/2014/294017

**Published:** 2014-03-04

**Authors:** J. Ma, L. Y. Liu, P. H. Wu, Y. Liao, T. Tao, W. Liu

**Affiliations:** ^1^Department of Endocrinology and Metabolism, Ren Ji Hospital, School of Medicine, Shanghai Jiao Tong University, No. 1630 Dongfang Road, Shanghai 200127, China; ^2^Department of Endocrinology and Metabolism, Shanghai Gongli Hospital, Shanghai, China

## Abstract

*Objective*. This study was designed to compare the effects of metformin and repaglinide on the fasting plasma glucose (FPG) and glycated haemoglobin (HbA1c) in newly diagnosed type 2 diabetes in China. *Methods*. A total of 107 newly diagnosed type 2 diabetic patients (46 women and 61 men) participated in the study. All patients received 3-month treatment of metformin or repaglinide. Fasting blood glucose and HbA1c were determined at baseline and at the end of the 3-month of treatment. *Results*. FPG and HbA1c decreased in both metformin and repaglinide groups after 3 months treatment (*P* < 0.01). The reduction of HbA1c was significantly greater in the repaglinide group (*P* < 0.01). Metformin decreases fasting insulin concentration and HOMA-IR (*P* < 0.01), and repaglinide improves HOMA-β  (*P* < 0.01). Triglycerides (TG) were reduced in both groups (*P* < 0.01 in metformin group; *P* < 0.05 in repaglinide group), but total cholesterol (TC) and low-density lipoprotein (LDL) were decreased only after metformin treatment (*P* < 0.05). *Conclusions*. Both repaglinide and metformin were effective in glycaemic control in new onset patients with type 2 diabetes in China. Repaglinide had no effect on insulin sensitivity, but it improved *β*-cell function.

## 1. Introduction

With the rapid economic growth and changes of lifestyle in the past few years, diabetes has become a worldwide epidemic in the general population. The prevalence of diabetes is up to 11.6% according to the data released in 2013 by the China Noncommunicable Disease Surveillance Group [1]. It is followed by higher associated morbidity and mortality. Glucose disregulation is a major risk factor for cardiovascular disease [2], which has become the leading cause of death [3]. In fact, the management of blood glucose in patients with type 2 diabetes in China is far from ideal [4–6]. Less than 40% of all diagnosed diabetes cases were well controlled [1].

According to the AACE Comprehensive Diabetes Management Algorithm 2013, the entry HbA1c is below 7.5%; patients with new onset type 2 diabetes should be on monotherapy following the lifestyle modification [7]. Among all kinds of antidiabetic medications, metformin inhibits hepatic glucose output and increases peripheral glucose uptake and utilization. Due to its blood glucose-lowering efficiency, beneficial effects on body weight, and protective effect on the cardiovascular system [8], it is recommended as the first-line antihyperglycaemic management for type 2 diabetes [9–13]. It also benefits patients with diabetes in the improvement of insulin sensitivity. However, most effects of metformin originated from studies in Caucasian population. There are few data coming from Asian patients with type 2 diabetes.

In newly diagnosed Chinese patients with type 2 diabetes, the decreased insulin secretion, in particular the first phase of insulin secretion, is the main characteristic [14]. When the fasting glycaemia of the type 2 diabetic patients was above 7.0 mmol/L, the acute insulin release was substantially reduced [15]. Optimization of glycaemic control represents a major aim in the management of diabetes. Furthermore, it is important that strategies normalize glycaemia without increasing the risk of hypoglycaemia. Repaglinide belongs to the meglitinide class with benzoic acid in its structure [16]. It decreases blood glucose level by stimulating the release of insulin in a rapid-acting style. Therefore, repaglinide is one of the most common antidiabetic medications in Chinese patients with higher postprandial glycaemia. Due to the complementary mechanism of metformin and repaglinide, the fixed dose of repaglinide and metformin has been used in the treatment of type 2 diabetes [17]. However, there were few studies comparing the effects of these two medications on glycaemic control in new onset Chinese patients with type 2 diabetes.

The study therefore aimed to compare the effects of metformin and repaglinide monotherapy on fasting blood glucose, HbA1c, body mass index (BMI), and lipids in the newly diagnosed Chinese patients with type 2 diabetes.

## 2. Methods

### 2.1. Subjects

All participants with type 2 diabetes, diagnosed by World Health Organization criteria, were recruited from the outpatient of the Endocrinology Department in Shanghai Renji Hospital. None had a history of coronary heart disease, abnormal renal function, active liver disease, chronic metabolic acidosis (including diabetic ketoacidosis), or severe chronic gastrointestinal disease. All of them were on two-week diet control before the enrollment.

The study protocol was approved by the Human Research Ethics Committee of the Shanghai Renji Hospital, and each patient was provided written informed consent. All studies were carried out in accordance with the Declaration of Helsinki and Good Clinical Practice guidelines.

### 2.2. Protocol (Table 1)

All patients were given diet and lifestyle advice and asked to keep behaviour modification programme as a part of study. 107 patients were divided into two groups randomly. 54 patients (24 women, 30 men; age: 56.4 ± 1.6 years; BMI 26.15 ± 0.46 kg/m^2^; HbA1c 6.95 ± 0.12%) received metformin 750–1500 mg/day, and 53 patients (22 women, 31 men; age 57.8 ± 1.3 years; BMI 24.45 ± 0.33 kg/m^2^; HbA1c 7.42 ± 0.13%) received repaglinide 0.75–1.5 mg/day. The administration of metformin or repaglinide was adjusted according to the blood glucose levels in the first week (FPG higher than 7 mmol/L or postprandial glucose more than 10 mmol). During this phase, the doses of all patients were optimized and they were followed by a 3-month treatment period. Adverse events were evaluated according to the Medical Dictionary for Regulatory Activities (MedDRA version 14.0). If the patients had hyperlipidaemia (TC > 5.7 mmol/L or LDL > 3.4 mmol/L), they would be given lipid-lowering drugs (Liptor 20 mg). There were 11 patients in the metformin group and 4 patients in the repaglinide group taking lipid-lowering drugs.

All participants presented to the Endocrinology Department in Shanghai Renji Hospital on two occasions, at “baseline” and after 3-month treatment period. On the evening before each study day, patients consumed a meal before 7 pm. After the meal, patients fasted from solids and liquids (14 h for solids, 12 h for liquids) until the following morning. On the study day, patients attended the department at 8 am and an intravenous catheter was inserted for blood sampling. Blood samples were collected for measurement of the fasting blood glucose, HbA1c, and lipids. Body weight and height were measured at baseline and at the end of treatment. Compliance was monitored by assessing a checklist which the subject marked after taking the medication and checked by weekly telephone contact.

### 2.3. Measurements

Blood glucose concentrations were measured by the hexokinase method; plasma insulin concentrations were assayed by radioimmunoassay kit (Beijing Atom HighTech Co., Ltd., Beijing, China). The intra-assay coefficients of variation (CV) of insulin were 5.5%. HbA1c levels were measured by high pressure liquid chromatography; lipid profile parameters were determined on a clinical chemistry analyser (Roche Original Reagents, Stockholm, Sweden).

### 2.4. Statistical Analysis

Blood glucose and serum insulin concentrations were analyzed over fasting periods at baseline and after 3-month treatment. The data are reported as means ± standard error. Statistical comparisons were made using independent *t*-tests for values before and after 3-month treatment, and paired *t*-tests between groups with SPSS 16.0 and *P* values <0.05 were considered significant.

## 3. Results

All patients completed the study and there were no adverse effects.

### 3.1. Fasting Blood Glucose Concentrations (Figure 1(a))

Fasting blood glucose was decreased after 3-month therapy both in the metformin (7.17 ± 0.15 mmol/L versus 6.29 ± 0.11 mmol/L, *P* < 0.01) and repaglinide groups (7.72 ± 0.17 mmol/L versus 6.46 ± 0.14 mmol/L, *P* < 0.01). There was no significant difference in reduction of fasting blood glucose between two groups.

### 3.2. HbA1c (Figure 1(b))

Significant improvements in HbA1c were seen in both metformin (6.95 ± 0.12% versus 6.34 ± 0.08%, *P* < 0.01) and repaglinide treatment groups (7.42 ± 0.13% versus 6.28 ± 0.09%, *P* < 0.01). HbA1c was decreased greater in the repaglinide group (*P* < 0.01).

### 3.3. Fasting Plasma Insulin (Figure 1(c))

In metformin group, fasting plasma insulin levels were significantly decreased from 11.95 ± 1.00 IU/L to 9.09 ± 0.74 IU/L after 3 months of treatment (*P* < 0.01). Fasting plasma insulin levels were 8.30 ± 0.53 IU/L at baseline and the values tended to be higher (9.64 ± 0.92 IU/L) after 3-month repaglinide treatment. Insulin resistance index (HOMA-IR) is fasting insulin (mU/mL) × fasting plasma glucose (mmol/L)/22.5. HOMA-IR is strongly related to insulin resistance. HOMA-IR decreased significantly during 3 weeks in metformin group (3.92 ± 0.38 versus 2.58 ± 0.22, *P* < 0.01), but it was not different after repaglinide treatment (2.92 ± 0.22 versus 2.86 ± 0.30, *P* = 0.851). Insulin sensitivity index (ISI) is 1/(FPG × FINS). ISI is useful for measuring insulin sensitivity. Similarly, ISI was reduced in metformin group (−4.29 ± 0.08 versus −3.88 ± 0.08, *P* < 0.05); but it was not different in repaglinide group (−4.05 ± 0.07 versus −3.92 ± 0.10, *P* = 0.159). However, *β*-cell function was measured by HOMA-*β*. HOMA-*β* is an index of insulin secretory function derived from fasting plasma glucose and insulin concentrations: HOMA-*β* = FINS × 20/(FPG − 3.5). It was improved in repaglinide group (40.28 ± 2.41 versus 67.65 ± 6.08; *P* < 0.05), but the improvement was not found in metformin group (67.38 ± 5.03 versus 68.80 ± 5.84, *P* = 0.81).

### 3.4. BMI (Figure 1(d))

In metformin group, BMI was significantly decreased after 3 months of treatment (26.15 ± 0.46 kg/m^2^ versus 25.52 ± 0.46 kg/m^2^; *P* < 0.05). BMI was slightly decreased after treatment of repaglinide (24.45 ± 0.33 kg/m^2^ and 24.30 ± 0.33 kg/m^2^); but the reduction of BMI was not statistically significant.

### 3.5. Serum Lipids

Significant decreases in TG were found in both the metformin and the repaglinide groups during three months of treatment (*P* < 0.01 and *P* < 0.05 resp.). However, improvements in TC and LDL were only found in the metformin group (*P* < 0.05). No changes of HDL-C were observed in either treatment group (Table 2).

## 4. Discussion

We showed that both metformin and repaglinide significantly decreased fasting blood glucose and HbA1c in newly diagnosed type 2 diabetes in China. As the studies in western countries, metformin was associated with increased HOMA-IR and ISI, while repaglinide improved *β*-cell function. The antidiabetic effect of repaglinide was greater than metformin, but the improvement of TC and LDL was only observed in metformin group.

The United Kingdom Prospective Diabetes Study (UKPDS) has demonstrated good glycaemic control as evaluated by HbA1c, is related to the lower risk of micro- and probably macrovascular complications of diabetes [18–20]. The contribution of fasting and postprandial hyperglycaemia to different levels of HbA1c is various [21, 22]. The study indicated that postprandial blood glucose levels play a primary role in overall glycemic exposure with HbA1c being below 7.3% [21]. The fasting blood glucose concentrations are important only when HbA1c increased over 10.2% [21]. However, recent study demonstrated that, for those patients with type 2 diabetes who required intensive hyperglycemic control, the contribution of fasting blood glucose to overall hyperglycemic exposure was fairly high (around 78%) with HbA1c range from 7.0% [22]. Furthermore, fasting hyperglycaemia is more frequent than postprandial hyperglycaemia in the prevalence of diabetes in China [1]. Therefore, we evaluated the fasting blood glucose levels and HbA1c in therapy-naive patients with type 2 diabetes.

Compared to Caucasians, Asian type 2 diabetic patients are characterised by impaired *β*-cell function rather than insulin resistance [23]. Therefore, insulin secretagogues are still one of the most widely used antidiabetic medications in China. Repaglinide is mainly used as a medication to lower postprandial hyperglycaemia. It has been demonstrated that the effect of repaglinide on fasting blood glucose is similar to the sulfonylureas with less hypoglycaemia occurrence [24]. Metformin decreases gluconeogenesis and glycogenolysis in the liver to lower basal blood glucose. In the current study, both metformin and repaglinide were effective in FPG reduction in newly diagnosed type 2 patients. After 3 months of treatment, there was no statistic difference in decrease of FPG between repaglinide and metformin. However, HbA1c reduction with repaglinide was significantly greater compared with that with metformin. Although we did not measure the postprandial glucose, we would speculate that a more marked reduction in postprandial glucose after repaglinide treatment at least partly contributes to the further decrease of HbA1c.

Repaglinide stimulates insulin secretion by closing ATP-dependent potassium channels and opening of calcium channels in the beta cell in response to ingestion. As a short-action insulin secretagogue, it mainly increases the first phase of insulin secretion, which is involved in suppression of hepatic glucose output and glucagon secretion [25]. It has been reported that repaglinide relieves the glucose toxicity to reduce fasting and postprandial insulin releases [26]. Thus, both HOMA-IR and HOMA-*β* were improved significantly during 12 weeks of treatment with repaglinide [27]. We confirmed that *β*-cell function has been improved in repaglinide group, but there was no improvement in HOMA-IR after repaglinide treatment. The different levels of fasting blood glucose should be considered. In our study, HbA1c and fasting blood glucose were comparably lower (7.7 ± 0.2 mmol/L) compared to the previous data in Italy (8.5 ± 1.3 mmol/L) [26]. Furthermore, we observed the insulinotropic effect in three months and the data might be changed on the long term. The parameters of *β* cell function were different with metformin. It is not surprising that metformin increases insulin sensitivity, expressed as HOMA-IR and ISI; but it has no effect in improving islet *β* cell function.

Lipidaemia represents an independent risk of cardiovascular diseases for patients with diabetes. The lowering effects of oral antihyperglycaemic medications on lipid profile and body weight should bring extra benefit for patients with type 2 diabetes. A study in China reported that repaglinide improves the lipid metabolism by reduction of TC in newly diagnosed patients with type 2 diabetes [28]. However, our study did not show any differences in TC or LDL after repaglinide dosage, although BMI and TG were decreased after 3 months. In conclusion, for overweight or obese type 2 diabetes patients, repaglinide can be considered as one of selecting antidiabetic medications. It is consistent with Chinese guideline for type 2 diabetes mellitus in 2010 [29].

This study has some limitations. Firstly, mean BMI of patients with type 2 diabetes in metformin group were higher than those in repaglinide group. However, BMI in both groups were in range of overweight (24.0–27.9 kg/m^2^) in Asian population. Moreover, it has been reported that the effects of metformin on glycaemic response were similar among normal weight (BMI <24 kg/m^2^), overweight, and obesity in Chinese patients with new onset type 2 diabetes [30]. Secondly, we did not measure postprandial blood glucose or plasma insulin concentrations after 3-month therapy just because repaglinide is well known for the prandial glucose regulation, but there was no study about the effects on fasting blood glucose in Chinese population. Thus, we focused on the effects of metformin and repaglinide therapy on fasting blood glucose in our study. Finally, the duration of this study was relatively short. Longer term studies are required in the future study.

In conclusion, this study demonstrated for the first time that both repaglinide and metformin monotherapies were effective in fasting blood glucose and HbA1c reduction, with the effect of repaglinide being greater in new onset Chinese patients with type 2 diabetes. Repaglinide improved *β*-cell function after 3-month treatment. However, the risk-predictive parameters of cardiovascular disease such as TC and LDL-C were only improved in metformin group.

## Figures and Tables

**Figure 1:**
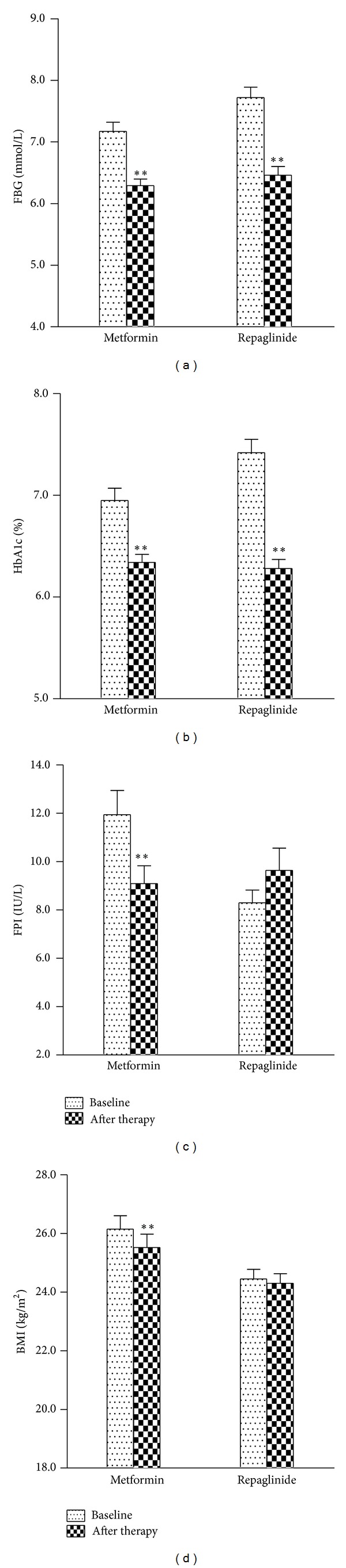
(a) Fasting blood glucose, (b) HbA1c, (c) fasting plasma insulin, and (d) BMI in 107 patients with type 2 diabetes before and after 3-month treatment of metformin or repaglinide. Data are means and standard errors. *P < 0.05; **P < 0.01.

**Table 1:** The characteristic of 107 participants (54 in metformin group and 53 in repaglinide group) with type 2 diabetes, diagnosed according to World Health Organization criteria.

Parameters	Group metformin			Group repaglinide		
	Men	Women	Both	Men	Women	Both
Gender	30	24	54	31	22	53
Mean age	55.4 ± 2.2	57.5 ± 2.4	56.4 ± 1.6	56.9 ± 1.7	59.1 ± 2.1	57.8 ± 1.3
BMI	26.18 ± 0.57	26.11 ± 0.78	26.15 ± 0.46	24.80 ± 0.43	23.96 ± 0.50	24.45 ± 0.33
HbAlc	7.11 ± 0.18	6.76 ± 0.13	6.95 ± 0.12	7.38 ± 0.16	7.48 ± 0.22	7.42 ± 0.13
FPG	7.28 ± 0.23	7.04 ± 0.17	7.17 ± 0.15	7.72 ± 0.20	7.72 ± 0.31	7.72 ± 0.17
FPI	11.36 ± 1.08	12.69 ± 1.81	11.95 ± 1.00	8.17 ± 0.75	8.49 ± 0.73	8.30 ± 0.53
TC	5.38 ± 0.17	5.25 ± 0.18	5.32 ± 0.12	4.98 ± 0.16	5.75 ± 0.23	5.30 ± 0.14
TG	2.11 ± 0.20	1.77 ± 0.15	1.96 ± 0.13	1.97 ± 0.20	2.14 ± 0.26	2.04 ± 0.16
HDL	1.26 ± 0.10	1.28 ± 0.06	1.27 ± 0.06	1.23 ± 0.05	1.51 ± 0.15	1.35 ± 0.07
LDL	3.34 ± 0.13	3.28 ± 0.14	3.32 ± 0.09	3.18 ± 0.16	3.48 ± 0.24	3.30 ± 0.13

Data are shown by means and standard errors. It was only different in TC between men and women of repaglinide group (*P* < 0.05).

**Table 2:** Serum lipids are compared before and after metformin or repaglinide treatment.

Parameters	Before treatment	After treatment
Group metformin		
TG (mmol/L)	1.96 ± 0.13	1.65 ± 0.11**
TC (mmol/L)	5.32 ± 0.12	5.02 ± 0.11*
HDL-C (mmol/L)	1.27 ± 0.06	1.26 ± 0.04
LDL-C (mmol/L)	3.32 ± 0.09	3.04 ± 0.09*

Group repaglinide		
TG (mmol/L)	2.04 ± 0.16	1.79 ± 0.14*
TC (mmol/L)	5.30 ± 0.14	5.16 ± 0.13
HDL-C (mmol/L)	1.35 ± 0.07	1.29 ± 0.04
LDL-C (mmol/L)	3.30 ± 0.13	3.20 ± 0.11

Data are means and standard errors. *P < 0.05; **P < 0.01.
